# SCHIZOPHENIA: DEFAULT MODE NETWORK CONNECTIVITY AS A FUTURE BIOMARKER

**DOI:** 10.1192/j.eurpsy.2023.2277

**Published:** 2023-07-19

**Authors:** M. A. L. Magalhães, M. Andrade

**Affiliations:** Centro Hospitalar Psiquiatrico de Lisboa (CHPL), Lisboa, Portugal

## Abstract

**Introduction:**

The Default Mode Network (DMN) is a brain system with physiological and cognitive properties that make it a major pillar of cortical integration. It has been a subject of increased research on different psychiatric conditions such as schizophrenia. It was hypothesized that alterations in the brain connectivity of the DMN, at the level of its functional connectivity (FC) and structural connectivity (SC), may be at the basis of this pathology. Thus, the DMN has been associated with several clinical variables such as symptom severity, disease prognosis and response to antipsychotic treatment, making this system a potential future tool in the study of these variables for better clinical guidance in patients with schizophrenia.

**Objectives:**

The aim of this study is to review the role of DMN in the pathophysiological mechanisms underlying schizophrenia, as well as its potential role as a future biomarker in detecting patients at high risk of developing a first psychotic episode and predicting therapeutic response to antipsychotics.

**Methods:**

Systematic review of the literature published on Pubmed using the terms: “Default Mode Network”, “Schizophrenia”, “First Psychotic Episode” and “Antipsychotics”.

**Results:**

A myriad of studies revealed the presence of dysfunctional DMN brain activity in patients with schizophrenia. However, increased FC of the DMN is the predominant outcome reported by literature in patients with and without chronic exposure to antipsychotic therapy, at high risk of developing psychosis and on both early and advanced disease stages, suggesting that the DMN may have a meritorious role on the pathophysiology of schizophrenia. Some studies have found SC changes associated with altered FC on patients at early stages of the disease without exposure to prolonged antipsychotic therapy. Regarding the relationship between DMN and antipsychotic therapy, studies suggested that DMN is shaped by antipsychotic therapy by regulating FC activity.

**Conclusions:**

This work helped us to understand the importance of the future study of the connectivity of the DMN in a longitudinal perspective of the course of schizophrenia in order to potentiate the creation of brain signatures that might translate the alterations of the connectivity of the DMN in early stages of the disease, which in turn could work as potential future biomarkers for the detection of patients at high risk of developing a first psychotic episode but also work on predicting the therapeutic response to antipsychotics, allowing us to direct our clinical orientation towards a better prognosis of the disease.

**Disclosure of Interest:**

None Declared

